# Weighted gene co-expression network analysis to define pivotal modules and genes in diabetic heart failure

**DOI:** 10.1042/BSR20200507

**Published:** 2020-07-07

**Authors:** Weiwei Liang, FangFang Sun

**Affiliations:** 1 Department of Endocrinology, The Second Affiliated Hospital, Zhejiang University School of Medicine, Hangzhou, P. R. China; 2 Department of Colorectal Surgery and Oncology, Key Laboratory of Cancer Prevention and Intervention, Ministry of Education, The Second Affiliated Hospital, Cancer Institute, Zhejiang University School of Medicine, Hangzhou, P. R. China

**Keywords:** Diabetes, Diabetic Heart Failure, WGCNA

## Abstract

This research was carried out to reveal specific hub genes involved in diabetic heart failure, as well as remarkable pathways that hub genes locate. The GSE26887 dataset from the GEO website was downloaded. The gene co-expression network was generated and central modules were analyzed to identify key genes using the WGCNA method. Functional analyses were conducted on genes of the clinical interest modules via Kyoto Encyclopedia of Genes and Genomes (KEGG) pathway and Gene ontology (GO) enrichment, associated with protein–protein interaction (PPI) network construction in a sequence. Centrality parameters of the PPI network were determined using the CentiScape plugin in Cytoscape. Key genes, defined as genes in the ≥95% percentile of the degree distribution of significantly perturbed networks, were identified. Twenty gene co-expression modules were detected by WGCNA analysis. The module marked in light yellow exhibited the most significant association with diabetes (*P*=0.08). Genes involved in this module were primarily located in immune response, plasma membrane and receptor binding, as shown by the GO analysis. These genes were primarily assembled in endocytosis and phagosomes for KEGG pathway enrichment. Three key genes, *STK39, HLA-DPB1* and *RAB5C*, which may be key genes for diabetic heart failure, were identified. To our knowledge, our study is the first to have constructed the co-expression network involved in diabetic heart failure using the WGCNA method. The results of the present study have provided better understanding the molecular mechanism of diabetic heart failure.

## Introduction

Heart failure (HF) is a widespread disease among persons over 65 years old. Heart failure can be manifested as difficulty in breathing and fatigue. It lowers the quality of life of the elderly and affects longevity. It is a chronic disease that requires lifelong management. The risk of HF is significantly high for individuals with diabetes mellitus (DM), compared with those without diabetes. Epidemiological evidence has shown the relationship between HF and DM. According to Nichols’s research, heart failure occurs in 30.9 per 1000 person years for diabetic patients and 12.4 per 1000 person years for non-diabetic patients [1]. Therefore, diabetes is recognized as a powerful independent risk factor contributing to death of hospitalized HF patients [2]. DM and HF often co-occur with each other, and moreover, intensify the risk for each other. A Danish investigation group, DIAMOND, found that 50% of HF patients with DM died within 3 years of follow-up. Mortality analyses showed a higher 1-year mortality (31%) in DM patients, compared with non-DM patients [2].

In diabetic patients, heart failure has been found to be promoted by inflammation, oxidative stress and hyperglycemia at the systemic level [3], while knowledge of the same is limited at the cellular and molecular levels. Therefore, current guidelines recommend the same therapy options for patients either with or without diabetes. Some randomized controlled trials have reported that, as a classical anti-diabetic medicine, the symptoms of HF patients may be alleviated by taking sodium-glucose co-transporter 2 (SGLT2) inhibitor [4,5]. Research data show that in diabetes patients who had failure, the use of SGLT2 inhibitors reduces hospitalizations and all-cause mortality due to heart failure compared to other hypoglycemic drugs [4,5]. However, other anti-diabetic drugs did not produce similar effects on HF even though they may exert the same anti-diabetic effect [6,7]. The beneficial effect on HF seems to be independent of the anti-diabetic effect caused by SGLT2 inhibition. Little is known on its molecular mechanisms. Whether SGLT2 inhibitors can protect those heart failure patients without diabetes is still unclear. Future clinical and basic experimental research are needed. Therefore, we aimed to identify the different core molecular mechanisms entangled in heart failure development with or without DM.

The biological function of cells is achieved through the interaction between genes to form a complex regulatory network. Genes with similar expression patterns may be co-regulated or functionally related. Gene network analysis is a method to find key genes by dividing genes into different modules by the similarity of expression, which helps us systematically understand the gene function at the molecular level. Weighted gene co-expression network analysis (WGCNA) has generally been implemented to effectively investigate associations among genome and clinical phenotypes [8]. WGCNA allows for genes to be clustered as co-expression units to determine the connection between characteristics of samples and differential gene expression. Thousands of genes can be analyzed and the corresponding gene modules for the clinical characteristics are identified through WGCNA to determine disease pathway related key genes for further validation. Systemic-level insights of the associated signaling network related to a particular phenotype are provided by WGCNA.

This research was implemented to reveal diabetes linked specific hub genes and pathways involved in heart failure. The GEO database was used to obtain rich data, while construction of the gene co-expression network, selection of important modules and identification of key genes was carried out using WGCNA. The results of this study have provided better understanding of the molecular mechanism involved in diabetic heart failure.

## Methods

### Data sources and searches

The Gene Expression Omnibus (GEO) is the largest source of publicly available microarray data. High-throughput functional genomic experiments on heart failure were searched for on GEO. For the present study, the selection criteria applied were: (1) studies containing data on heart biopsy samples of diabetic heart failure and non-diabetic heart failure patients; (2) studies on expression profiling by array; (3) raw data or processed data with public accessibility; (4) studies conducted on Homo sapiens; and (5) a total sample size of >15. The GSE26887 dataset was selected for the WGCNA analysis, because it was determined to be of the highest quality and has been conducted on an appropriate sample size.

### Data and statistical analysis

The GSE26887 dataset was obtained from the GEO website. Quality control, preprocessing and statistical analysis were performed using the limma package in Rstudio. The data are normalized through Robust Multi-array Average (RMA) method. WGCNA analysis was conducted on the entire GSE26887 dataset.

### WGCNA network construction and identification of modules

The WGCNA package was adopted to build the co-expression network [8]. First, the samples were clustered to identify obvious outliers. Second, the co-expression network was constructed using the automatic network construction function. Adjacency was calculated from the soft thresholding power *β*, which was derived by co-expression similarity, using pick-Soft-Threshold function. Third, modules were detected through hierarchical clustering and dynamic tree cut function. Fourth, for the modules correlated to the clinical attributes, module membership (MM) and gene significance (GS) were calculated. Information on genes in the modules were used for further analysis. Finally, the eigengene network was visualized.

### Functional enrichment analysis

Further functional enrichment analysis was applied on genes of the module of interest. Characteristic biological attributes of the genes were identified using GO analysis [9], while functional attributes were identified using KEGG pathway enrichment analysis [10]. A *P* value of < 0.05 was considered to show that a result was statistically significant.

### PPI network and identification of key genes

Protein–protein interaction (PPI) networks can provide information on the functional interactions between proteins. The construction of PPI network was based on the modules of interest by a joint of the Search Tool for the Retrieval of Interacting Gene (STRING) [11]. The protein interaction relationship network was visualized using Cytoscape [12].

Centrality parameters of the PPI network, including degree, closeness and betweenness, were determined using the CentiScape plugin [13] in Cytoscape. The average number of interactions relevant to each node was used as the degree of that particular node. Key genes, defined as genes in the ≥ 95% percentile of the degree distribution of clinically relevant networks, were identified.

## Results

### Microarray data acquisition and gene expression analysis

The GSE26887 dataset was further analyzed in the present study. GSE26887 contained microarray data on left ventricle cardiac biopsies of 7 diabetic and 12 non-diabetic post-ischemic heart failure patients. The raw data were downloaded on the GPL6244 platform from the GEO database of the National Center of Biotechnology Information (NCBI). Information on the clinical characteristics of the samples were also retrieved from the GEO database.

The RMA method in the limma package was used for raw data normalization. Then, genes with the highest average expression values ranked before 4000 were enrolled for further evaluation. All genes and samples were found to have an adequate number of values available. Next, the samples were clustered to locate any obvious outliers. A value of 25 was set as the cut-off height and samples that did not meet this criterion were removed (Supplementary Figure S1).

### Construction of the gene co-expression modules

**Figure 1:**
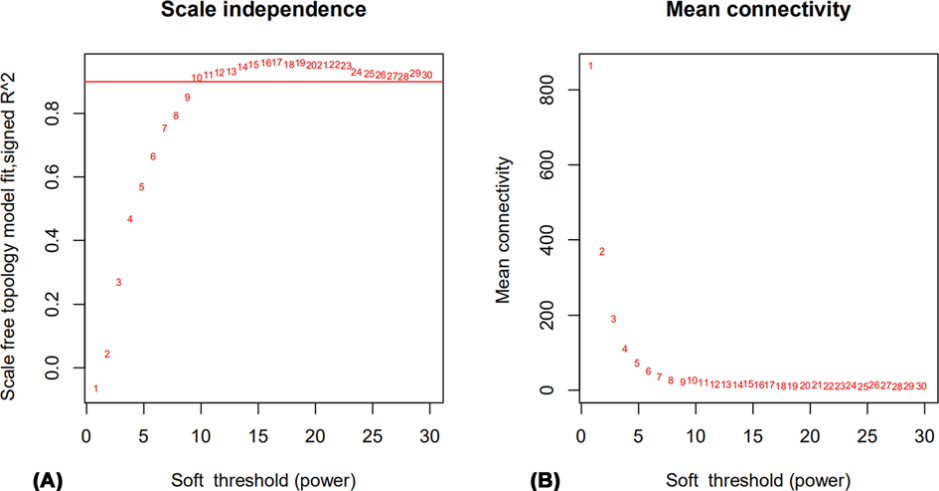
Selection of the soft-thresholding powers based on analysis of network topology (**A**) *X* axis represents soft-thresholding power. The *Y* axis represents the scale-free topology model fit index. (**B**) *X* axis represents soft-thresholding power. The *Y* axis represents the mean connectivity (degree).

The soft thresholding power *β*, a parameter derived from the co-expression similarity and aimed to determine neighborhood, was first calculated for the construction of the WGCNA network. The pick-Soft-Threshold function in WGCNA was used to analyze network topology. A value of 10 was chosen as the soft thresholding power *β* value for the next analysis because the level of independence was able to reach 0.9 (Figure 1A) and average connectivity was relatively high (Figure 1B).

**Figure 2:**
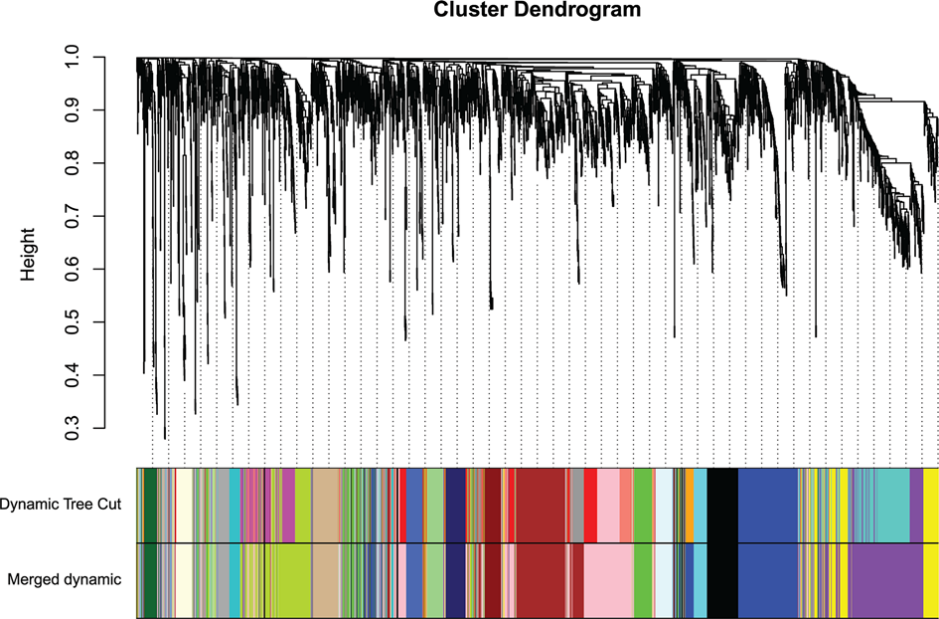
Clustering dendrogram of genes The dendrogram of clustering is based on topological overlap. Genes with similarity is clustered in one module color.

Using WGCNA, the gene network was constructed and its one-step network construction function used to identify modules. A value of 30 was chosen as the minimum module size and a value of 2 was chosen as the deepSplit value (which indicates a medium level of sensitivity). In total, 20 gene co-expression modules (Figure 2 andTable 1) were detected and constructed.

**Table 1:** Number of genes in constructed modules

Module color	Number of genes
module black	159
module blue	300
module brown	290
module cyan	94
module dark green	61
module dark gray	57
module dark red	66
module dark turquoise	60
module green	166
module green yellow	242
module gray	58
module light cyan	88
module light green	69
module light yellow	67
module midnight blue	90
module pink	403
module purple	383
module royal blue	67
module tan	105
module yellow	107

### Exploration of the pairwise gene co-expression modules and the eigengenes

Relationships among the established modules were dug deep and mapped (Supplementary Figure S2). The Topological Overlap Matrix (TOM) of the 4000 genes on which the analysis was performed is shown on the heatmap. A lighter color indicates a lower level of overlap, while the darker the color the higher the level of overlap. The results indicated relative independence between the modules.

Key gene (eigengene) connectivity was analyzed before the key genes were clustered. Eigengenes may be used to elucidate the association between pairwise gene co-expression modules. The results showed that the 20 modules could be assigned to two distinct clusters. Each cluster contained 10 modules (Supplementary Figure S3A). As shown in Supplementary Figure S3B, there were four combinations (brown and royal blue modules, black and cyan modules, purple and yellow modules, and green and light cyan modules) that showed a high degree of interconnectivity.

**Figure 3:**
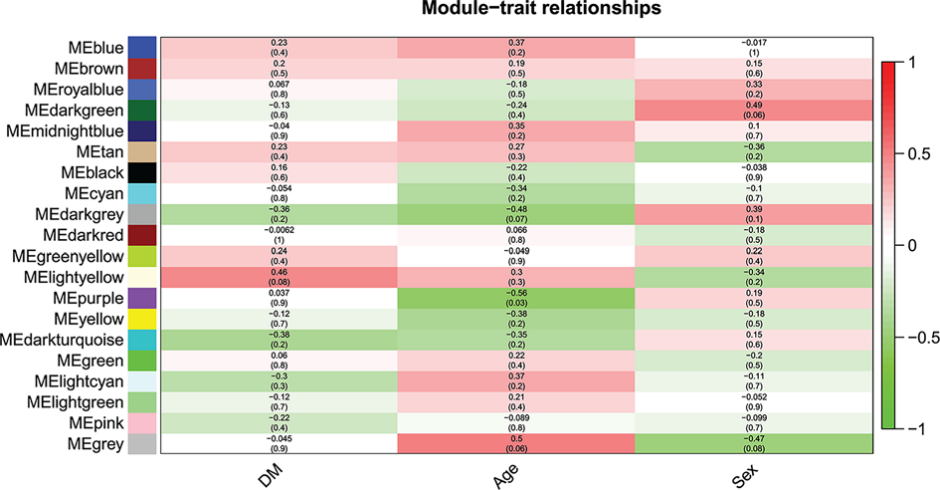
The associations between module and trait Each row represents to a specific module, and the column corresponds to a specific trait. The value of each cell represents the corresponding correlation and *P*-value. Different colors represent the degree of correlation.

### Identification of the key module

The correlation between the modules and the clinical features was analyzed to determine associations with the highest levels of significance. The results showed that the light yellow module was most significantly correlated with diabetes (*P*=0.08) (Figure 3).

### Key module functional analysis

**Figure 4:**
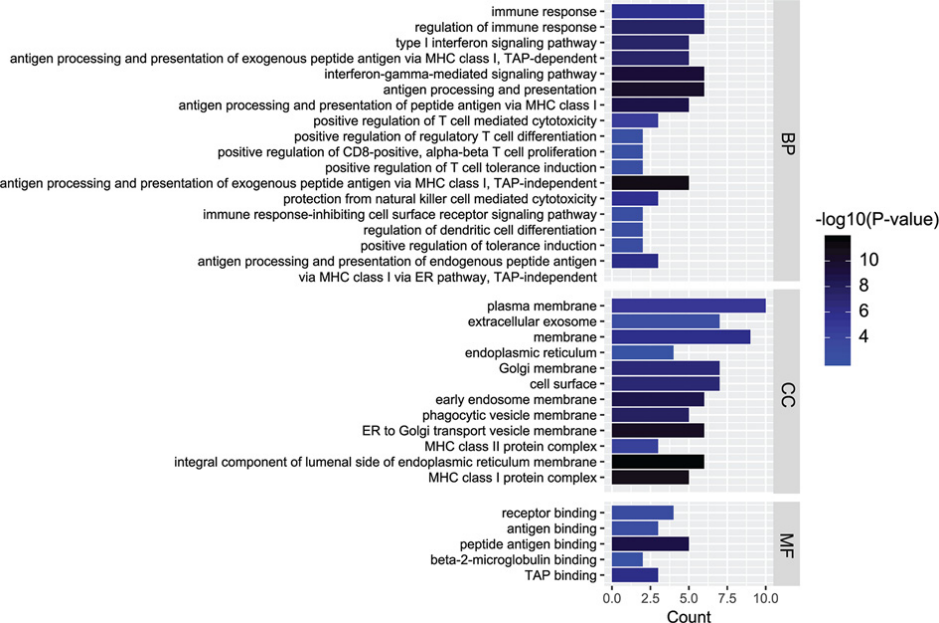
Gene ontology analysis Gene ontology analysis for biological process, cellular component and molecular function of genes involved in module light yellow. Immune response was the most enriched biological process, the plasma membrane was the most enriched cellular component, while receptor binding was the most enriched molecular function among the genes.

**Figure 5:**
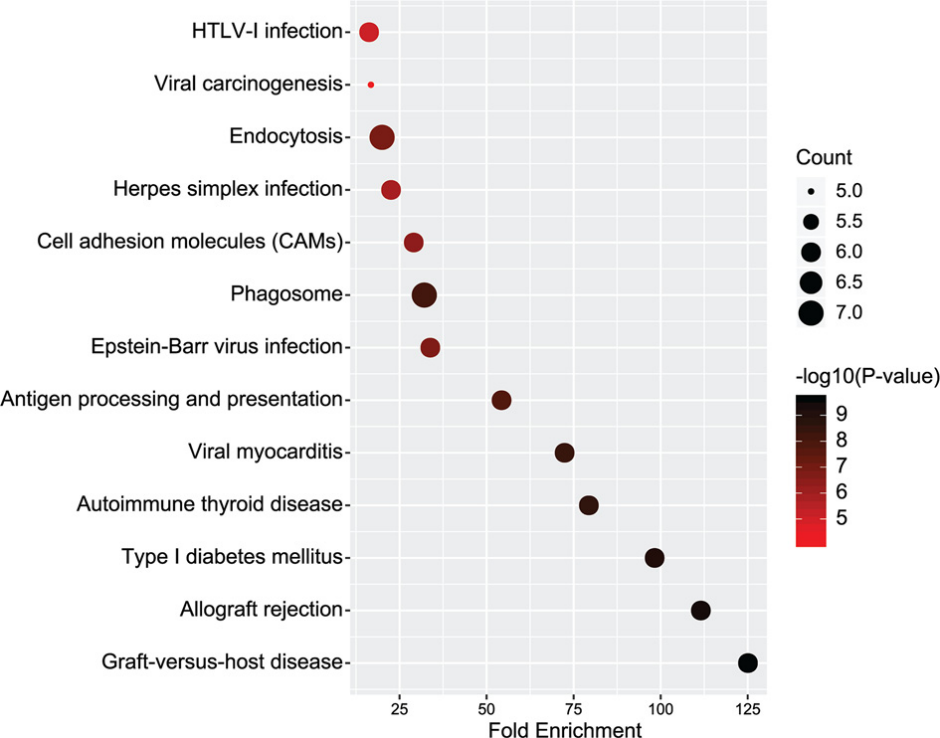
Kyoto Encyclopedia of Genes and Genomes analysis of genes involved in module light yellow Different node size reflects the gene count. The larger node means more gene counts. Different node color reflects *P* value [− log10(*P* value)]. The darker the node color, the greater the *P* value is.

Genes in the light yellow module, which was most significantly correlated with diabetic heart failure, were managed with further functional analysis, including KEGG enrichment and GO analysis (Figures 4 and 5). The chief pathways, in which genes in the key module were mainly enrolled in, were determined as endocytosis and phagosomes, through the KEGG analysis. As for GO analysis, immune response was the most enriched biological process, the plasma membrane was the most enriched cellular component, while receptor binding was the most enriched molecular function among the genes.

### Construction of PPI network

**Figure 6:**
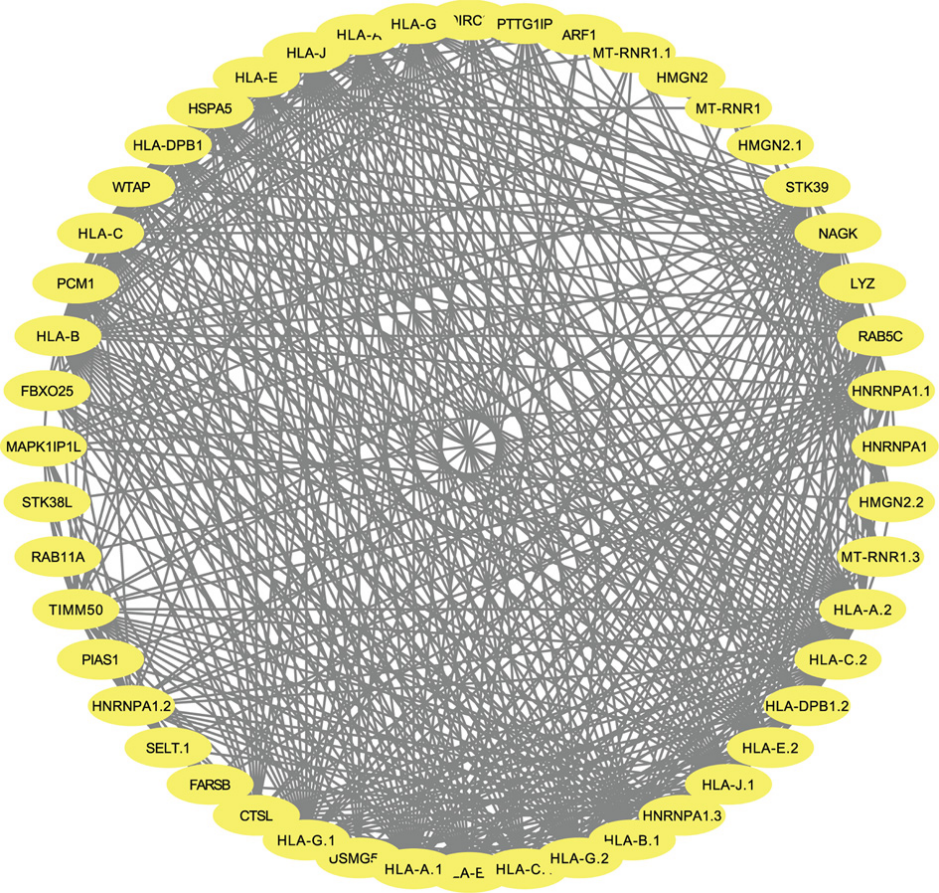
PPI network PPI network of genes involved in module light yellow generated by the Cytoscape software. STRING was applied to create PPI network to define protein interactions.

STRING was applied to create PPI network to define protein interactions (Figure 6). Using the CentiScape plugin, the key genes were identified as *STK39, HLA-DPB1* and *RAB5C* (Table 2).

**Table 2:** Degree, closeness and betweenness of key genes in the PPI network

Gene	Degree	Betweenness	Closeness
*STK39*	22	17.14	0.01
*HLA-DPB1*	22	1.42	0.01
*RAB5C*	21	2.52	0.01

## Discussion

Heart failure and diabetes are common clinical diseases. Prognosis of diabetic heart failure patients is poor and the life expectancy is short. Patients with diabetic heart failure often cause a greater disease burden. Therefore, it is of great clinical significance to find molecular mechanism of diabetic heart failure, and may be the promising targets for future treatment of diabetic heart failure.

In this research, key modules and genes involved in diabetic heart failure were observed using bioinformatics methods. Using published diabetic heart failure data, we detected and constructed co-expression modules in WGCNA and identified the key modules. Functional analysis and protein–protein interaction networks of the key modules were investigated. Finally, *STK39, HLA-DPB1* and *RAB5C* were defined as key genes.

WGCNA, a systemic approach, is frequently used to explore pairwise connections among enormous genes in transcriptional level [8]. Unlike other network analysis methods concentrating on unweighted networks, WGCNA is more comprehensive and qualified to create correlation networks no matter weighted or unweighted [8]; therefore, the results of the analysis are reliable and significant. WGCNA groups functionally related genes into a module, therefore biologically relevant modules can be classified for further analysis. As far as we are concerned, this was the first research to explore underlying causation of diabetic heart failure by constructing the co-expression network based on WGCNA method, and to better investigate diabetic heart failure related genes.

In the present study, through the deep and systemic reanalysis of the GSE26887 dataset, the light yellow module was determined to be the most significant for diabetic heart failure. GO analysis demonstrated that immune response was activated during the development of diabetic heart failure. This result is consistent with previous findings on heart failure [14]. Studies have shown that activation of the immune response provokes adverse cardiac remodelling and causes left ventricular dysfunction [14,15]. There are also studies that have shown that the immune response is activated during the development of diabetes [16]. The crossover of immune response may be a trigger for the progression of diabetic heart failure, which is worthy of further study. The KEGG analysis showed that endocytosis and phagosomes were important pathways involved in diabetic heart failure. Endocytosis and phagosomes are intimately allied to autophagy, which is concerned to participate in the pathologic progress of many diseases, including diabetes [17]. Autophagy process disorder often results in cardiomyopathy and cardiac dysfunction [18,19]. Revealing the physiological processes of endocytosis and phagosomes could highlight possible targets that potentially contributes to the treatment of diabetic heart failure as well as the prevention.

In our research, three key genes were found to be involved in diabetic heart failure. *STK39*, belonging to serine/threonine kinase family, is recognized as a dominant factor in the cellular stress response pathway. Only a few studies have revealed the specific function of *STK39* on diabetic heart failure. *STK39* was recognized as a hypertension risk factor through whole-genome association studies [20,21]. Klooster’s study showed that *STK39* was associated with insulin resistance in humans, which is the key pathological process of diabetes [22], while Hafver’s study showed that STK39 could bind to NCX1, a sodium–calcium exchanger involved in end-stage human heart failure [23]. These studies have shown that *STK39* is worthy of further investigation. *HLA-DPB1* encodes a major histocompatibility complex that present peptides derived from extracellular proteins and casts a vital function on immune system. Previous studies have shown that *HLA-DPB1* is associated with diabetes and dilated cardiomyopathy [24,25], therefore deeper investigation of *HLA-DPB1* in diabetic heart failure is required. *RAB5C* is a protein coding gene, belonging to the Rab protein family. Boutchueng–Djidjou’s recent study showed that *RAB5C* is an endosomal sorting marker that is associated with diabetes. However, only a finite number of investigations have revealed the potential mechanism how *RAB5C* functions in the development of diabetic heart failure. Therefore, further exploration is needed to delineate the essential biological processes through which *RAB5C* alters the progression of diabetic heart failure.

The limitations of our study need to be noted. The construction of the correlation network is only a preliminary analysis and phenotype-specific networks, such as transcription factors-gene networks, were not identified through our study [26]. Therefore, further studies using larger sample sizes, as well as functional studies on key genes involved in diabetic heart failure are required. Molecular biology methods should be used to validate our finding. Further *in vivo* and *in vitro* experiments may be done to verify the results of the present study.

In conclusion, our research was the first study to explore underlying causation of diabetic heart failure by constructing the co-expression network based on WGCNA method. The present study identified key genes that can be further investigated to determine the molecular mechanisms underlying diabetic heart failure.

## Supplementary Material

Supplementary Figures S1-S3

## Data Availability

The raw data of GSE26887 was available from GEO website (https://www.ncbi.nlm.nih.gov/geo/query/acc.cgi?acc=GSE26887).

## Abbreviations

GO: Gene ontology
KEGG: Kyoto Encyclopedia of Genes and Genomes
PPI: protein–protein interaction
RMA: Robust Multi-array Average
