# Supplementary material for: Weighted gene co-expression network analysis to define pivotal modules and genes in diabetic heart failure
Source: Biosci Rep. 2020 Jul 7;40(7):BSR20200507. doi: 10.1042/BSR20200507 (PMC7340867; doi:10.1042/BSR20200507)
Supplement: Supplementary Figures S1-S3 [file BSR-2020-0507_supp.pdf]

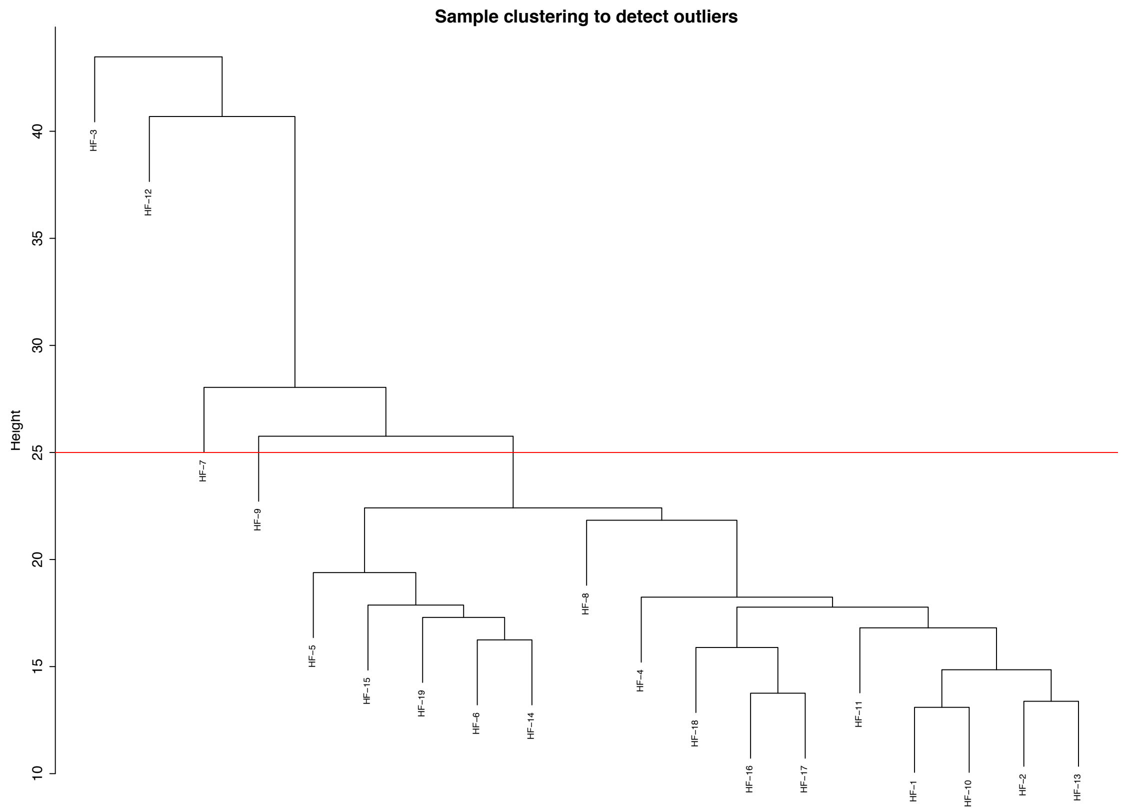

Sup. Figure 1 Clustering dendrogram of samples based on their Euclidean distance.

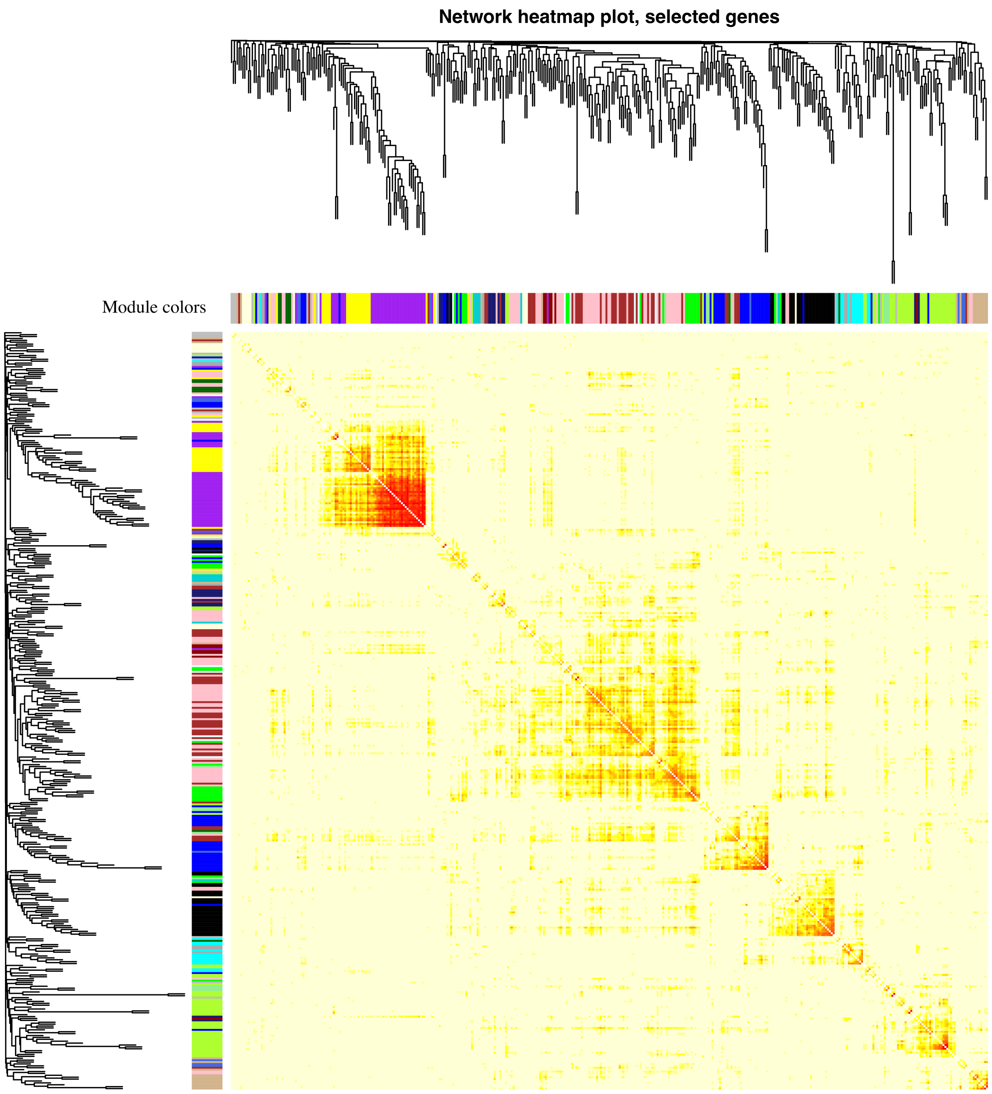

Sup. Figure 2 visualizing WGCNA network using a heatmap plot. The heatmap depicts the topological overlap matrix (TOM) among all modules in the analysis. The colors mean different modules. Light color represents low overlap and progressively darker red color represents higher overlap.

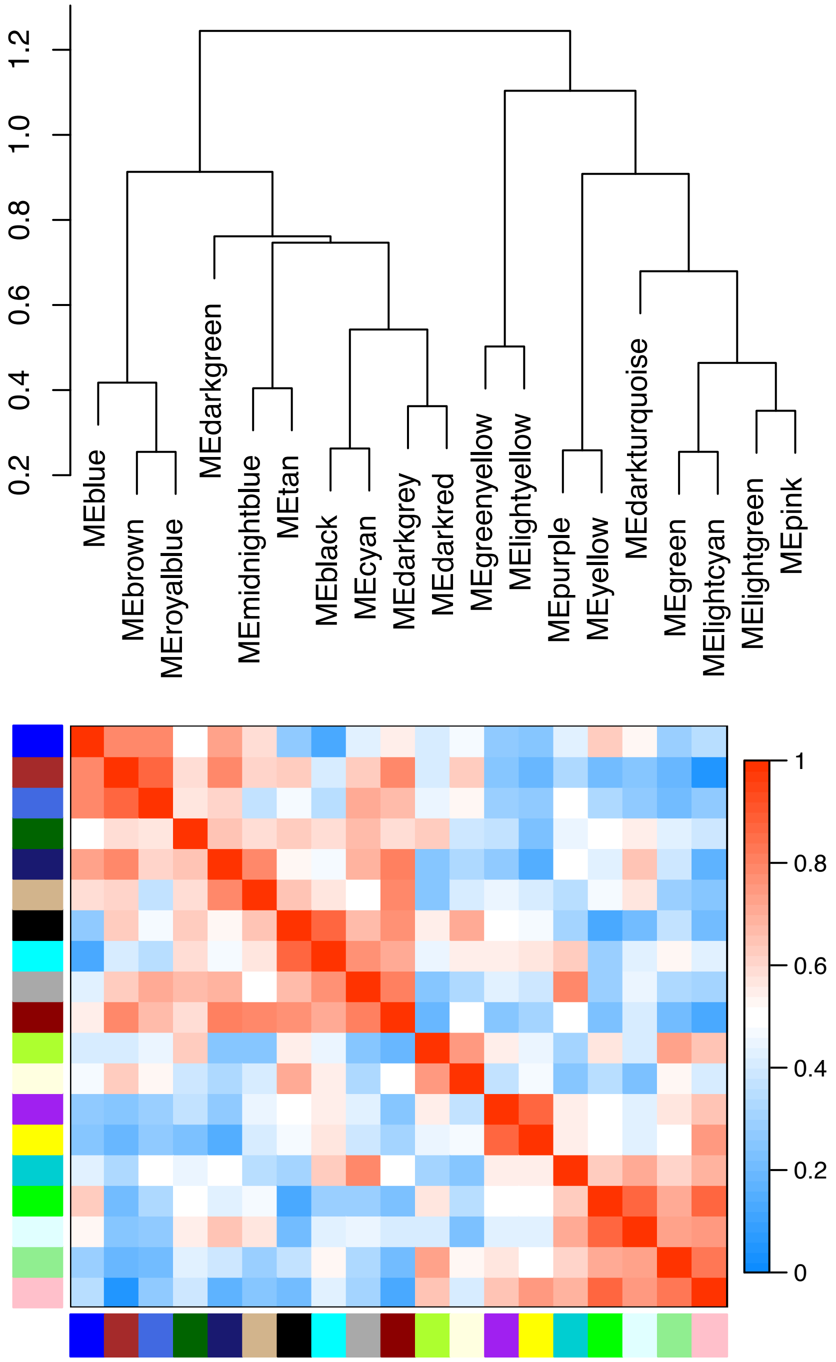

Sup. Figure 3 Eigengene dendrogram and eigengene adjacency plot.
